# Brucellosis and its associated risk factors to humans and domestic ruminants in Kagera Ecosystem, Tanzania

**DOI:** 10.4314/ahs.v21i2.6

**Published:** 2021-06

**Authors:** Jean Bosco Ntirandekura, Lucas Eliaimringi Matemba, Sharadhuli Iddi Kimera, John Bwayla Muma, Esron Daniel Karimuribo

**Affiliations:** 1 Sokoine University of Agriculture, College of Veterinary Medicine and Biomedical Sciences; 2 Université du Burundi, Département de Santé et Productions Animales; 3 National Institute for Medical Research, Dodoma, Tanzania; 4 University of Zambia, School of Veterinary Medicine, Department of Disease Control, Lusaka, Zambia

**Keywords:** Brucellosis, pastoralists, risk factors, Tanzania

## Abstract

**Background:**

Brucellosis is an important disease for both veterinary and public health. A study was conducted to understand the seroprevalence of brucellosis and its associated risk factors in pastoral areas of Kagera, Tanzania.

**Methods:**

Sera from 156 patients with malaria-like symptoms were analyzed using the commercial rapid agglutination test (specific for *B.abortus* and *B.melitensis* detection) and Fluorescence Polarization Assay (FPA). Sera from 426 cattle, 206 goats and 197 sheep were analyzed using Rose Bengal Plate (RBPT) and Competitive ELISA (c-ELISA) tests.

**Results:**

In humans, overall brucellosis, *B. abortus*, and *B. melitensis* sero-prevalences were 7.7% (95%CI: 3.8–12.2%), 1.9% (95% CI: 0.4–4.5%), and 5.8 % (95%CI: 2.6–10.6%), respectively. At animal level, seropositivity was 5.9% (95%CI: 4.0–8.6%), 2.5% (95%CI: 0.8–5.7%) and 0.5% (95%CI: 0.01–2.8%) in cattle, goats and sheep, respectively. At herd level, seropositivity was 18.2% (95%CI: 12.0–25.8%) in cattle and 6.9% (95%CI: 2.2–15.3%) in small ruminants. Brucellosis was associated with assisting in parturition without wearing protective gears (OR= 5.6; p= 0.02) in humans, herds of 50–200 animals (OR= 4.2, p= 0.01) and cattle (OR=3.5; p=0.01). The knowledge of brucellosis among pastoralists (OR=0.1; p<0.01) was a protective factor.

**Conclusion:**

Brucella infections could be occurring in pastoralists and domestic ruminants in Kagera. Community health education is necessary for the control of brucellosis in Tanzania.

## Introduction

Brucellosis is a zoonotic disease that affects humans and animals globally. It is acquired by direct or indirect contact with infected animals or their products. In humans, the disease is under-diagnosed worldwide 1 and the symptoms are often vague but may include: undulating fever (the most common symptom), body-wide aches and pains, headache, and night sweats. Domestic animals are infected through a direct contact with aborted materials, vaginal discharges, milk and semen from Brucella infected animals. Different studies on brucellosis have been carried out in East African countries. In Uganda 2 and Kenya 3 studies reported different prevalence levels, depending upon the locations, the methods of diagnosis used and according to species.

Previous studies on brucellosis in Tanzania have demonstrated the importance of the disease as zoonosis^4^–^7^. Other studies conducted on brucellosis in some ecosystems in Tanzania reported prevalence of this disease in humans, livestock and wildlife interface. For instance, anti-Brucella antibodies were detected in humans (0.6 %); in cattle (6.8 %), in goats (1.6 %) and in buffaloes (7.9 %) 4. In addition, a 10.5% prevalence of brucellosis in trade stock of Karagwe district has been reported, which poses a risk of its transmission through trade animals 8. Due to its economic importance and social impact in the population (miscarriages, infertilities and reduction of milk production), brucellosis in Kagera ecosystem calls for researchers' attention. This region is in an ecosystem that borders with three countries (Burundi, Rwanda and Uganda) where, domestic animals, wildlife and human populations are constantly interacting 9. Studies on brucellosis prevalence in this shared ecosystem between bordering countries are scarce. However, such researches could underscore the understanding of the transboundary issues associated with the disease transmission and the movement of people and teir livestock within ecosystems in East African Community (EAC). Therefore, the objective of this study was to estimate the magnitude of Brucella infection and identify associated risk factors among pastoralists and their domestic ruminants in Kagera ecosystem, Tanzania.

## Methodology

### Study design

**Figure 1:**
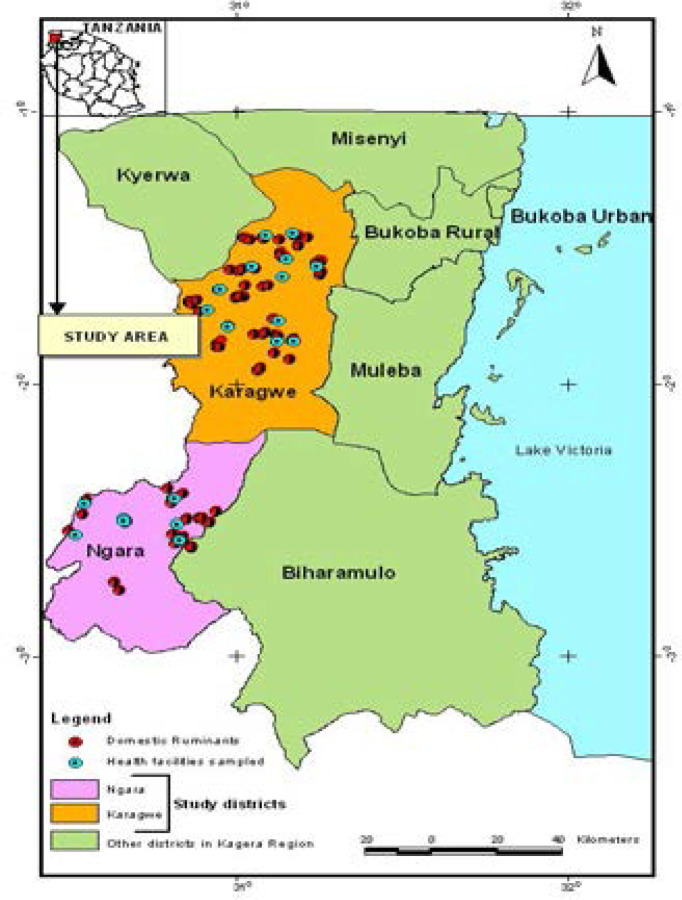
Map showing study districts (Karagwe and Ngara)

A cross-sectional study was conducted in June 2017 to identify risk factors associated with seropositivity against Brucella spp. in humans and domestic ruminants in pastoral areas of Kagera Region (two districts: Ngara and Karagwe). Eighteen villages were purposively selected from peri-urban and rural areas (Figure 1).

Ten herds were also purposively selected in each village after considering some factors like villages located on borders with neighboring countries, herds with mixed animals, herds practicing communal grazing. Moreover, health facilities (dispensaries and health centers) located in the selected villages were included in the study for blood sampling on patients with malaria-like symptoms (fever, joint pain, headache, back pain, fatigue and nausea). In addition, two district hospitals were included for human sampling due to the big number of patients frequenting in these areas. Participants were recruited in health facilities (14 health centers and 5 hospitals) at the moment they were coming for malaria checking in the morning. Most patients that were included in the study lived in close proximity with domestic animals that were also sampled in this study area and majority of them (94%) were animal keepers. Assisted by local phlebotomists, plain vacutainer tubes were used to collect 5ml of venous blood from patients. Prior to this, consent was obtained after explaining the study objectives to the participants. Using a formula: Z2* Pexp (1 -Pexp) /D2 10, sample sizes of 234 humans, 492 cows, 200 goats and 200 sheep were calculated. Herds were grouped according to the number of animals (small size: 1–50 animals; medium size: 50–200 animals and large: >200 animals). Domestic ruminants were randomly sampled in the selected households using plain vacutainer tubes to collect 5ml of venous blood assisted by local veterinary technicians. Sampling was done after getting the owner's consent. Interviews were conducted on patients and owners of animals in Kiswahili (national language). Due to the language barrier of the principal investigator, an appointed translator assisted. Structured questionnaires digitalized by AfyaData software 11 were filled using smart mobile phones to collect data on risk factors with main variables in patients (symptoms, consumption of unboiled milk, assisting parturition without wearing protective gears, living in close proximity with domestic animals, livestock keeping). In case of domestic ruminants, factors evaluated included: sharing source of water, communal grazing, sharing bulls and history of miscarriage.

### Laboratory analysis

Human sera were double checked for anti-Brucella antibodies of *B. abortus* and *B. melitensis* or for cross-reactions using the commercial rapid agglutination test according to the manufacturer's instructions (ARKRAY Healthcare Pvt.ltd-INDIA, lots 15SA402-05 and 15SA403-05). Only samples with agglutinations on the slides were subjected to the confirmation using Fluorescent Polarization Assay according to the manufacturer's instructions (Ellie LLC, USA, Brucella FPA, code B1001). The cut-off was fixed at 115 mP (millipolarization units) with a sensitivity and specificity of 99.03 and 99.9%. For domestic animals, sera were screened firstly using Rose Bengal Plate test. Samples only with a reaction were then subjected to confirmation using the c-ELISA test according to manufacturers' instructions (APHA Scientific, UK. COMPELISA 160&400). Patients (humans) reacting to Rapid slide and FPA tests were considered to be seropositive to brucellosis. Domestic ruminants were considered to be seropositive if they reacted to both RBP and c-ELISA tests.

### Ethical consideration

This study was also approved by institutional review board of Sokoine University of Agriculture and the Medical Research Coordinating Committee of the National Institute for Medical Research (ref: NIMR/HQ/R.8a/Vol.IX/2456).

### Data analysis

Questionnaires were uploaded in excel sheet for analysis. Seroprevalence in humans and domestic ruminants were determined based on criteria of seropositivity for an individual during laboratory analysis. The overall seroprevalence were determined for each species and the herd seroprevalence was also computed. All independent variables were screened by univariable logistic regression analysis for their association with the positivity of brucellosis in Kagera using IBM® SPSS® Statistics 21. Variables with p-value less than 0.2 (univariable logistic regression) were included in the risk factors assessment by a multivariable logistic regression model, reporting odds ratio with 95% confidence intervals. A P-value less than 0.05 was considered as significant.

## Results

### Demographic characteristics of humans and ruminants sampled in Kagera Region

A total of 192 herds were visited and 156, 426, 206 and 197 sera were sampled from humans, cattle, goats and sheep, respectively. Patients were aged between 5 and 77 years (mean =35±16.6) with a total of 55 males and 101 females (sex ratio of 1:2) in the two districts. Livestock owners were between 24 and 84 years of age (mean = 47.5 ± 11.7), and the age of animals sampled was between 18 months and 8 years for cattle, and 8 months to 5 years for small ruminants.

### Seroprevalence of brucellosis in humans and domestic ruminants in Kagera Region

In humans, from FPA test, the overall brucellosis (n=12), *B. abortus* (n=3), and *B. melitensis* (n=9) sero-prevalences were 7.7% (95%CI: 4.04 – 13.05%), 1.9% (95%CI: 0.4 – 4.5%), and 5.8 % (95% CI: 2.6–10.6%), respectively. No sample was positive to both *B. meltensis* and *B. abortus*. The overall seroprevalence of brucellosis in domestic ruminants (from c-Elisa test) was 3.7% (95% CI: 4 – 8.6) while the herd prevalence was 13.5% (95% CI: 9 – 19.2%). At herd level, the prevalence of brucellosis was 18.2% (95% CI: 12–25.8%) and 6.8% (95% CI=2.2–15.3%) in cattle and small ruminants, respectively.

### Logistic regressions

The univariable logistic regression was done to assess the association between each variable and the seropositivity of brucellosis in Kagera region (Table 1 & Table 2). The final model for the risk factors associated to Brucella infections in humans and domestic ruminants are presented in Table 3.

**Table 1:** Univariable logistic regression between positivity of brucellosis and different variables in humans in Kagera Region

Variable	Extent	N	Positive to FPA test (%)	p-value
District	Ngara	53	5 (9.4)	0.56
	Karagwe	103	7 (6.8)	

Health facilities	Local dispensaries	113	9 (7.9)	0.8
	District hospitals	46	3 (6.9)	

Sex	Female	97	8 (8.2)	0.98
	Male	59	4 (6.8)	

Age	5–30 years	74	5 (6.7)	0.6
	31–60 years	62	5 (8)	
	>60 years	20	2 (10)	

Marital status	Single	36	1 (2.8)	0.6
	Married	110	11 (10)	

	Divorced	4	0 (0)	
	Widower	6	0 (0)	

Fever	Yes	133	10 (7.5)	0.8
	No	23	2 (8.7)	

Back pain	Yes	62	3 (4.8)	0.4
	No	94	9 (9.6)	

Joint pain	Yes	40	4 (10)	0.7
	No	116	8 (7)	

History of miscarriage (women)	Yes	5	1 (20)	0.3
	No	151	11 (7.3)	

Dinking unboiled milk	Yes	70	8 (11.4)	0.2
	No	86	4 (4.7)	

Consuming cheese	Yes	33	4 (12.1)	0.47
	No	123	8 (6.5)	

Living with domestic animals	Yes	120	9 (7.5)	0.84
	No	36	3 (8.3)	

Assisting parturition without protective gears	Yes	19	4 (21.1)	0.06
	No	137	8 (5.8)	

History of miscarriage (herd)	Yes	116	9 (7.8)	0.7
	No	40	3 (7.5)	

Poor disposal of aborted materials in herds	Yes	78	9 (11.5)	0.1
	No	78	3 (3.8)	

Permanent contact with livestock	Yes	148	11 (7.4)	0.87
	No	8	1 (12.5)	

**Table 2:** Univariable Logistic regression of risk factors and the seroprevalence of brucellosis in domestic ruminants (animal level) in Kagera Region

Variable	Extent	N	Positive to c- Elisa test (%)	p- value
District	Karagwe	580	27 (4.7)	0.05
	Ngara	249	4 (1.6)	

Species	Cattle	403	6 (1.5)	<0.01
	Caprine and ovine	426	25 (5.9)	

Herd management	Pastoralism	735	30 (4.1)	0.2
	Zero grazing	50	0 (0)	
	Mixing	44	1 (2.3)	

Herd size	Medium (50–200)	500	26 (5.2)	0.02
	Small (<50)	329	5 (1.6)	

Herd location	Rural	747	29 (3.9)	0.72
	Peri-urban	82	2 (2.4)	

Communal grazing	Yes	169	9 (5.3)	0.32
	No	660	22 (3.3)	

Sharing pasture with wildlife	Yes	247	13 (5.3)	0.19
	No	582	18 (3.1)	

Sharing water sources among herds	Yes	573	23 (4.0)	0.67
	No	256	8 (3.1)	

Sharing bulls among herds	Yes	237	5 (2.1)	0.12
	No	592	26 (4.4)	

History of miscarriage (in herds)	Yes	601	26 (4.3)	0.21
	No	228	5 (2.2)	

Knowledge on brucellosis among pastoralists	Yes	367	3 (0.82)	<0.01
	No	462	28 (6.06)	

Vaccination of diseases (in herds)	Yes	602	28 (4.7)	0.04
	No	227	3 (1.3)	

Vaccination of brucellosis (in herds)	Yes	62	1 (1.6)	0.4
	No	767	30 (3.9)	

**Table 3:** Multivariable logistic regression of risk factors of brucellosis positivity in humans and domestic ruminants in Kagera

Variables	Extent	OR	95% IC	p-value
**Risk factors in humans**				

Drinking unboiled milk	Yes	3.2	0.8–12.4	0.09
	No	Ref		

Assisting in parturition without protective gears	Yes	5.6	1.3–23.5	0.02
	No	Ref		

**Risk factors in domestic ruminants (herd level)**				

Species	Bovine	3.5	1.3–9.8	0.01
	Caprine and ovine	Ref		

Herd size	Small (<50)	Ref		
	Medium (50–200)	4.2	1.4–12.7	0.01

Knowledge on brucellosis among pastoralists	Yes	0.1	0.02–0.5	<0.01
	No	Ref		

## Discussion

Brucellosis is a zoonotic disease of concern for both veterinary and public health in Tanzania 12–14. In this study, a rapid agglutination test detected *B. melitensis* and *B. abortus* antibodies which were confirmed by the FPA test in patients with malaria-like symptoms. Our results are closer to those reported in a study conducted in Moshi, Northern Tanzania 5, but there are lower compared to reports from Sengerema district 15 and Mwanza Region 16. Nevertheless, there was no difference regarding positivity to brucellosis among febrile and non-febrile participants (Table 1). This may be due to the small number of persons who were seropositive to brucellosis in this group. In Mikumi ecosystem, the assessment of brucellosis prevalence in febrile group revealed higher positivity of 23.9% than in non-febrile group 3.7% 17. In addition, there was no difference of Brucella positivity between women with history of miscarriage and those witout such experience (Table 1). This could be associated to the low number of pregnant women in our sample. Although some studies have demonstrated the interactions between brucellosis and the animal trophoblast, the pathogenesis of miscarriage in women remains very unclear^18^. In other report in Tanzania, no prevalence of brucellosis was found in humans in agro-pastoral communities of Serengeti district 7. Elsewhere, seropositivity of brucellosis in humans were also reported in Uganda (17%) 19; in Kenya (2.2–14.1%) 20 and in Ethiopia (3–34.9%) 21. Assisting parturitions without wearing protective gears (Table3) increased the risk of exposure to Brucella infections in humans in Kagera. Similar results were reported in northern part of Tanzania^5^. In addition, the habit of drinking unboiled milk (Table 3) seemed to be a risk factor of for human infections even if it was less significantly associated with brucellosis seropositivity in Kagera. Furthermore, there is a report on habit to drink unboiled milk by people from the study area 22.

The prevalence of brucellosis has previously been detected in trade stock at 10.5% 8. Our results are similar to those reported in indigenous cattle (animal level 5.6% and herd level 21.7%) in western part of Tanzania^23^ and closer to those reported in Lushoto and Rungwe districts^24^ in small dairy cattle. In addition, the prevalence of brucellosis was 11.3% in indigenous cattle in Mbeya Region 25. Other studies reported prevalence of brucellosis in cattle 4,26 in pastoral areas of Tanzania. In this study, the prevalence of bovine brucellosis at herd level was within the range (16.2%, 95% CI: 10.2 -25.7%) reported in sub-Saharan Africa 27. In this study, cattle seemed to be more at risk of getting Brucella infections compared to small ruminants (Table 3). This could be due to the abundance of this species in this pastoralist area, but also to the susceptibility of cattle to different Brucella species which can be originated from infected domestic or wild animals commingled in the study area. Also, the herds with increased number of animals (50–200) were at risk to acquire Brucella infection (Table 3). Similar results were reported in Mbeya Region 25 also in other parts of Africa 28,29. In fact, animals in large herds of cattle are condemned to long mobility and migration for pasture and water sources which increase the risk of intermingling between domestic ruminants and favor the Brucella infection in pastoral areas 3. However, bovine brucellosis is persistent in livestock with low level and relative stability of transmission in pastoral areas^30^. In this study, the knowledge of brucellosis among pastoralists (Table 3) was a protective factor for the disease in domestic ruminants. Nevertheless, vaccination against diseases in the herds in general was not significant in the final logistic model. These results indicated that the knowledge of the disease among pastoralists should be complimented with the vaccination program and practices of good hygiene for controlling brucellosis in their herds as reported before in the study area 22.

## Study limitations

Due to financial limitations, this study couldn't extend the human sample size to the community in non-fever group which could have generated additional information for the understanding of seroprevalence of brucellosis in the study area. However, sampled patients lived in close proximity with domestic animals that were also sampled in this study and majority of them (94%) were animal keepers. The financial and time limitations influenced much the purposive sampling in this study which affects the generalizability of our results. Nevertheless, this contributed to understand the consistent of known animal husbandry and movement risk factors in the study area.

## Conclusion

The seropositivity of brucellosis is distributed according to susceptible species (humans, cattle, goat and sheep) located in pastoral area of Kagera. The identified main risk factors in humans for the persistence of brucellosis in Kagera were assisting in animal parturition without wearing protective gears while in domestic ruminants, herd size and cattle increased the risk of Brucella infection. However, the knowledge of brucellosis among pastoralists seemed to be a protective factor for this zoonotic disease. Further studies with advanced diagnostic methods (culture and molecular characterization) are suggested to assess this important zoonotic disease in Kagera Region. These results are proposed for use in understanding of brucellosis in Tanzania also for the implementation of control and surveillance of this disease in East Africa.
